# On Leucocythæmia, Considered as Specific Cancer of the Blood

**Published:** 1888-07

**Authors:** L. Bard

**Affiliations:** Hospital Physician and Chief of the Anat. Path. Laboratory of the Faculty of Medicine, Lyons


### Selections.

### *ON LEUCOCYTHÆMIA, CONSIDERED AS SPECIFIC CANCER OF THE BLOOD.*

By L. BARD, Hospital Physician and Chief of the Anat. Path. Laboratory of the Faculty of Medicine, Lyons.

Leucocythæmia, which is well to be distinguished from leucocythosis—the latter disappears with its causes, and, for this reason, is of short duration, while the former is always progressive, and always terminates with death—is the cancer of the blood, as epidermoidal epithelioma is the cancer of the skin. It is not the formation of a solid tumor that constitutes the characteristic of cancer, it is the increase of pathologic tissue—in the blood, that of the white corpuscula—its steady, irresistible progress from the beginning of the affection, foreign to the inflammatory process and leading to a specific cachexy, which always terminates in death. Leucocythæmia, it is true, is usually connected with hypertrophy of the spleen and of the lymphatic glands, as well as of the so-called hæmatopoetic organs, liver, marrow of the bones, intestinal follicles, thymus, accessory kidneys, and with the formation of small neoplasmata of the structure of the adenoid tissue in hypertrophied organs, but this is only an enormous dilatation of the capillary net-work, crammed with leucocytes. The other lymphoid lesions have for their starting-point vascular eruptions in consequence of stagnations, having led to real apoplexies of the white corpuscles of the blood. The modifications of the blood consist in: (1) Multiplication of the white corpuscles; (2) apparition of globules, *i. e.*, young embryonic forms of leucocytes; (3) considerable diminution of the red corpuscles. All these facts agree with the clinical image of cancer, while the classical hypothesis of the origin of leucocythæmia, excessive formation of white corpuscles of the blood in the hypertrophied organs, is refuted by the following circumstances: (1) Multiplication of the white corpuscles not unfrequently being met with without any hypertrophies being present; (2) multiplication of the white corpuscles mostly preceding hypertrophy in time; (3) the specific epithelium of the spleen, the marrow of bones, the lymphatic glands being different from the white corpuscles of blood histochemically as well as biologically. Moreover, this hypothesis has led various authors to the admission of lymphogenous diathesis or lymphatic adenitis, for the clinical demonstration of which they have associated three diseases of entirely different nature: leucocythæmia, Trousseau's adenitis or Hodgkin's disease, and mycoses fungoides. Of these three diseases, it is only the Hodgkin disease which may combine different affection, it is to say the true cases pertaining to cancer of the lymphatic glands and to the specific infections varieties of adenitis.

What precedes demonstrates the clinical analogy of leucocythæmia with malignant tumors; the pathologico-anatomical analogy results from the following researches, part of which has been published by Bard in the last three years: (1) Never one tissue is transformed into another one, their specific cells producing only cells of the same nature; (2) *all* normal tissues of the human body may give rise to tumors of a mild or of a malignant character, so that an unlimited number of species of tumors is to be admitted, separable into malignant and mild ones. But, although tumors of all tissues may occasionally assume either a mild or a malignant character, it is demonstrated by facts that tumors of certain tissues will, as a rule, assume the malignant type, while tumors of other tissues, as a rule, are of a mild type (*Virchow*, diagn. and progn. of cancer, D. M. Z. 7188). Malignant tumors alone show the characteristic feature of steady and rapid growth through cellular multiplication, and of their structure being made up from embryonic cells which are still of young age. The forms pertaining to the latter in the different tissues, are not known as yet, and for this reason malignant tumors have been divided into different species, without any regard to their originating organs.

This definition of malignant tumors is eminently applicable to leucocythæmia, which is a cancer of the leucocytes, multiplying steadily and rapidly, and giving rise to embryonic forms, the globules. The fact that there are no tumors forming depends on the peculiarity of the blood tissue to be fluid, and, therefore, to produce fluid tissues only, and not solid tissues. The general symptoms and the cachety following cancer of the blood, is, as in all cancers, the exclusive result of hindrance or suppression of the functions of the tissue affected; the enormous number of new-formed cells acts, in this case, mechanically and through their biological properties. The white blood corpuscle is by no means identical with the lymphatic cell, and, in cancer of the blood, is not to be confounded with cancer of the lymphatic glands, leucocythæmia having no connection with the various classes of adenites. Leucocytosis, for its part, is to leucocythæmia as in solid tissues hyperplastic inflammation is to cancer.—*Deutsche Medizinal Zeitung.*

